# First Report of a Toxic *Nodularia spumigena* (Nostocales/ Cyanobacteria) Bloom in Sub-Tropical Australia. II. Bioaccumulation of Nodularin in Isolated Populations of Mullet (Mugilidae)

**DOI:** 10.3390/ijerph9072412

**Published:** 2012-07-05

**Authors:** Ian Stewart, Geoffrey K. Eaglesham, Glenn B. McGregor, Roger Chong, Alan A. Seawright, Wasantha A. Wickramasinghe, Ross Sadler, Lindsay Hunt, Glenn Graham

**Affiliations:** 1 Queensland Health Forensic and Scientific Services, 39 Kessels Road, Coopers Plains, Queensland 4108, Australia; Email: g.eaglesham1@uq.edu.au (G.K.E.); Lindsay_Hunt@health.qld.gov.au (L.H.); Glenn_Graham@health.qld.gov.au (G.G.); 2 School of Public Health, Griffith University, Parklands Drive, Southport, Queensland 4217, Australia; Email: ross.sadler@griffith.edu.au; 3 Environment and Resource Sciences, Queensland Department of Science, Information Technology, Innovation and the Arts, Ecosciences Precinct, Boggo Road, Dutton Park, Queensland 4102, Australia; Email: Glenn.Mcgregor@derm.qld.gov.au; 4 Biosecurity Queensland, Department of Agriculture, Fisheries and Forestry, 39 Kessels Road, Coopers Plains, Queensland 4108, Australia; Email: Roger.Chong@daff.qld.gov.au; 5 The University of Queensland, National Research Centre for Environmental Toxicology (EnTox), 39 Kessels Road, Coopers Plains, Queensland 4108, Australia; Email: a.seawright@uq.edu.au (A.A.S.); w.wickramasinghe@uq.edu.au (W.A.W.); 6 Faculty of Science, Health and Education, University of the Sunshine Coast, Sippy Downs Drive, Sippy Downs, Queensland 4556, Australia

**Keywords:** cyanobacteria, cyanotoxin, nodularin, *Nodularia*, seafood, mullet, recreational, HPLC-MS/MS

## Abstract

Fish collected after a mass mortality at an artificial lake in south-east Queensland, Australia, were examined for the presence of nodularin as the lake had earlier been affected by a *Nodularia* bloom. Methanol extracts of muscle, liver, peritoneal and stomach contents were analysed by HPLC and tandem mass spectrometry; histological examination was conducted on livers from captured mullet. Livers of sea mullet (*Mugil cephalus*) involved in the fish kill contained high concentrations of nodularin (median 43.6 mg/kg, range 40.8–47.8 mg/kg dry weight; *n* = 3) and the toxin was also present in muscle tissue (median 44.0 μg/kg, range 32.3–56.8 μg/kg dry weight). Livers of fish occupying higher trophic levels accumulated much lower concentrations. Mullet captured from the lake 10 months later were also found to have high hepatic nodularin levels. DNA sequencing of mullet specimens revealed two species inhabiting the study lake: *M. cephalus* and an unidentified mugilid. The two mullet species appear to differ in their exposure and/or uptake of nodularin, with *M. cephalus* demonstrating higher tissue concentrations. The feeding ecology of mullet would appear to explain the unusual capacity of these fish to concentrate nodularin in their livers; these findings may have public health implications for mullet fisheries and aquaculture production where toxic cyanobacteria blooms affect source waters. This report incorporates a systematic review of the literature on nodularin measured in edible fish, shellfish and crustaceans.

## 1. Introduction

Cyanobacterial hepatotoxins and neurotoxins have long been known to cause death and acute illness in wild animals and livestock. Mass intoxications have been documented for over a century; such incidents led to the understanding that cyanobacterial waterblooms could be poisonous [1]. *Nodularia spumigena* and its associated toxin nodularin also have a long history of poisoning stock animals and wildlife. Indeed, the world’s first detailed scientific description of mass mortality attributed to toxic cyanobacteria concerned exposure to *Nodularia*-contaminated water in Australia. Sheep, horses, dogs and pigs were killed [2]. Mass poisoning of ducks, cattle and dogs associated with exposure to *Nodularia* blooms in northern European waters has been reported [3,4]. 

The chemical structure of nodularin, a cyclic pentapeptide, was first described in 1988 [5], so earlier reports of hepatotoxic injury and death from exposure to *Nodularia* cannot definitively confirm nodularin as the responsible agent. However, as *Nodularia* is not known to produce toxins other than nodularin and some minor structural variants, it would seem reasonable to assume that nodularin was the cyanotoxin responsible for historical poisonings associated with exposure to waters contaminated by *Nodularia*.

**Figure 1:**
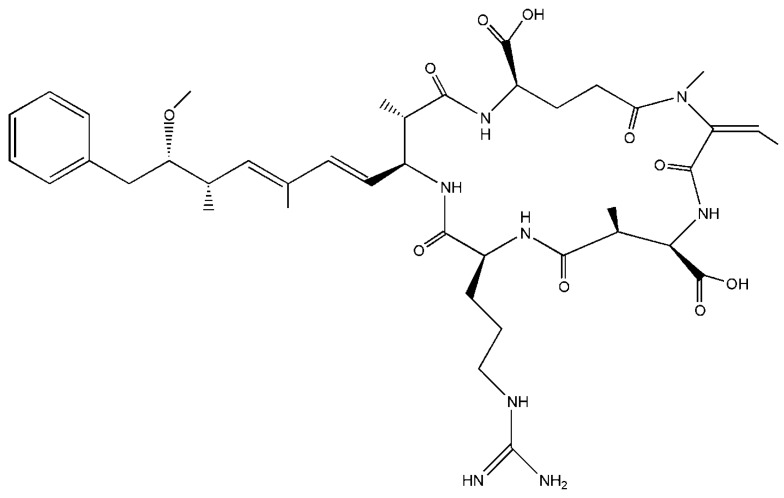
Structure of nodularin.

Following elucidation of the structure of nodularin (Figure 1) and the growing understanding of its toxicity, methods for identifying and quantifying the toxin in various biological matrices have been developed. Most current techniques are based on ELISA, HPLC, LC-MS and tandem mass spectrometric methods. 

The ability to identify nodularin in animal tissues has led to the recognition that this toxin (as well as other cyanotoxins) can be found in measurable quantities in animal products that are food items for humans. The potential for dietary transfer of nodularin to humans was initially investigated by Falconer *et al*. [6], who demonstrated lethal hepatotoxicity in mice exposed to boiled extracts of mussel viscera. *Nodularia* trichomes were identified in mussel gut samples, and nodularin was the presumptive cause of the pathological findings. The authors recommended that edible mussels should not be consumed when toxic cyanobacterial blooms are present in source waters. Since then, a series of research investigations into the presence of nodularin in edible tissues has been conducted. Most of this work comes from Finland; toxic *Nodularia* blooms are a regular occurrence in the Baltic Sea, and there is concern regarding the potential for nodularin to contaminate common northern European seafood and shellfish like flounder, herring, mussels and clams. 

This study was initiated after preliminary investigation of a fish kill in a small recreational lake in south-east Queensland revealed that nodularin concentrations in mullet liver were some three orders of magnitude higher than those found in carnivorous fish. We sought to widen the scope of our study by capturing mullet from the affected lake to ascertain whether tissue nodularin levels had resolved, and we identified nearby lakes that had not been subject to *Nodularia* blooms to source mullet for reference comparisons. In order to place our findings into context with work from elsewhere in the world on nodularin in edible fish, we conducted a systematic review of the literature on nodularin detected and quantified in seafood and shellfish. 

**Table 1:** Reported concentrations of nodularin in marine animal food products.

Species	Common name	Date collected	Matrix	Nodularin concentration ^#^ μg/kg	Method	LOD/LOR	References
*Platichthys flesus*	European flounder	1997–2000	muscle	ND	ELISA	0.2 ppb LOD	[7,8,9]
*Platichthys flesus*	European flounder	2007	muscle	ND	HPLC-UV	NR	[10]
*Mytilus edulis*	blue mussel	2007	whole soft tissues	ND	HPLC-UV	NR	[10]
NR	mussel	NR	whole soft tissues	0.2 ww	LC + PPIA	NR	[11]
*Platichthys flesus*	European flounder	2005	muscle	1.0 dw	ELISA + confirmatory LC-MS	0.1 ppb LOD	[12]
NR	finfish	2001	muscle	2.5 ww	LC-MS	NR	[13]
*Salmo salar*	Atlantic salmon	1997	liver	5 dw	ELISA	0.31 ppb IC_50_	[14]
*Clupea harengus membras*	Baltic herring	2002	liver	5 dw	LC-MS	NR	[15]
*Clupea harengus membras*	Baltic herring	1997	muscle	6.5 dw	ELISA	0.31 ppb IC_50_	[14]
*Salmo salar*	Atlantic salmon	2002	liver	10 dw	LC-MS	NR	[15]
*Somateria mollissima*	eider	2002	muscle	21 dw	LC-MS	NR	[16]
NR	prawn	2001	muscle	22 ww	LC-MS	NR	[13]
*Platichthys flesus*	European flounder	2001	liver	28 dw	LC-MS	5 pg	[17]
*Somateria mollissima*	eider	2002	liver	48 dw	LC-MS	10 pg on column	[18]
*Macoma balthica*	Baltic clam	2006 ^+^	whole soft tissues (gut rinsed)	52 dw (mean of *n* = 3 specimens)	LC-MS	NR	[19]
*Gadus morhua*	Atlantic cod	1998	liver	53 dw	ELISA	0.31 ppb IC_50_	[9]
*Mytilus edulis*	blue mussel	2005	whole soft tissues	80 dw	LC-MS	10 pg on column	[18]
*Sprattus sprattus*	sprat	2004	whole fish	100 dw	ELISA	NR	[20]
*Platichthys flesus*	European flounder	2004	muscle	100 dw	ELISA + LC-MS	NR	[16]
*Salmo trutta*	sea trout	NR ^+^	muscle	125 dw	ELISA	0.5 ppb	[21]
*Platichthys flesus*	European flounder	1997	liver	140 dw	ELISA	0.31 ppb IC_50_	[9]
*Mytilus edulis*	blue mussel	2004	whole soft tissues	139 dw	ELISA + confirmatory LC-MS	0.1 ppb LOD	[12]
NR	finfish	2001	viscera	152 ww	LC-MS	NR	[13]
*Somateria mollissima*	eider	2002	liver	180 dw (LC-MS SIR)	ELISA + LC-MS	NR	[22]
*Somateria mollissima*	eider	2002	liver	199 dw	LC-MS	NR	[16]
*Rutilus rutilus*	roach	2004	muscle	200 dw	ELISA + LC-MS	NR	[16]
*Mytilus edulis*	blue mussel	2004	soft tissues minus hepatopancreas	200 dw	LC-MS	50 pg on-column	[23]
*Clupea harengus membras*	Baltic herring	2004	whole fish	220 dw	ELISA	NR	[20]
*Platichthys flesus*	European flounder	NR ^+^	liver	220 dw	LC-MS		[24]
*Mytilus edulis*	blue mussel	2001	whole soft tissue	303 dw	LC-MS	5 pg	[17]
*Macoma balthica*	Baltic clam	NR	whole soft tissues	320 dw	HPLC + diode array	120 ppb LOQ	[25]
*Platichthys flesus*	European flounder	1999	liver	399 dw	ELISA + confirmatory LC-MS	0.2 ppb LOD	[7,8]
*Platichthys flesus*	European flounder	2000	liver	410 dw	ELISA	0.2 ppb LOD	[8]
*Platichthys flesus*	European flounder	2005	liver	473 dw	ELISA + confirmatory LC-MS	0.1 ppb LOD	[12]
*Platichthys flesus*	European flounder	2007	liver	557 dw	HPLC-UV	NR	[10]
*Platichthys flesus*	European flounder	1995	liver	637 ww	LC-MS (multiple reactant monitoring)	1–2 ppb (selected ion recording)	[26]
*Penaeus monodon*	black tiger prawn	2001/02	hepatopancreas	830 dw	ELISA + confirmatory HPLC + diode array	0.2 ppb (ELISA) 0.3–0.5 ng per injection	[27]
*Rutilus rutilus*	roach	2004	liver	900 dw	ELISA + LC-MS	NR	[16]
*Platichthys flesus*	European flounder	2004	liver	1,100 dw	ELISA + LC-MS	NR	[16]
*Mytilus edulis*	blue mussel	2004	hepatopancreas	1,100 dw	LC-MS	50 pg on-column	[23]
*Salmo trutta*	sea trout	NR^+^	liver	1,200 dw	ELISA	0.5 ppb	[21]
*Mytilus edulis*	blue mussel	1999	whole soft tissues	2,150 dw	ELISA + confirmatory LC-MS	0.2 ppb LOD	[7,8]
*Platichthys flesus*	European flounder	2002	liver	2,230 dw	ELISA + confirmatory LC-MS	0.2 ppb * LOD	[28]
NR	mussel	2001	whole soft tissue	2,500 ww	LC-MS	NR	[13]
NR	prawn	2001	viscera	6,400 ww	LC-MS	NR	[13]
*Mytilus edulis*	blue mussel	2002 ^+^	whole soft tissue	13,750 dw	LC-MS	NR	[29]
*Macoma balthica*	Baltic clam	2000 ^+^	whole soft tissue	30,300 dw 1,400 dw	ELISA HPLC + diode array	0.16 ppb 200 ppb LOQ	[30]
*Mytilus edulis*	blue mussel	NR ^+^	whole soft tissue	80,400 dw	LC-MS	0.5 ng (abs)	[31,32]
*Mytilus edulis*	blue mussel	NR ^+^	digestive gland	245,000 dw	LC-MS	0.5 ng (abs)	[31,32]

*: ELISA LOD reported as 0.2 g/L, presumably in error; ^+^: experimental exposure; ^#^: where a range of nodularin concentrations are reported, the maximum concentration is tabled here (except where noted); ND: nil detect; NR: not reported; dw: dry weight; ww: wet weight.

**Table 2:** Nodularin concentrations in tissues of specimens from the study recreational lake and nearby reference lakes.

Common name	Species	Location	Date specimen obtained	Tissue	Nodularin concentration, μg/kg wet weight. Median (range)	C.V. (%)	Nodularin concentration, μg/kg dry weight equivalents. Median (range)
sea mullet; *n* = 3	*Mugil cephalus*	recreational lake	November 2008	Muscle ^T^	13.6 (10.0, 17.6)	6.1	44.0 (32.4, 57.0)
				Liver ^T (*n* = 2) D (*n* = 1)^	12,000 (11,200, 13,200)	8.3	43,500 (40,600, 47,800)
				peritoneal contents ^T^	195 (132, 277)	8.3	
greasy rockcod; *n* = 1	*Epinephelus tauvina*	recreational lake	November 2008	Muscle ^T^	ND		
				Liver ^T^	25.2	10.7	91.3
longfin eel; *n* = 1	*Anguilla reinhardtii*	recreational lake	November 2008	Muscle ^T^	ND		
				liver	58.6		212
yellowfin bream; *n* = 2	*Acanthopagrus australis*	recreational lake	November 2008	Muscle ^T^	ND		
longfin eel; *n* = 1	*Anguilla reinhardtii*	recreational lake	April 2009	Liver ^T^	7.64	1.0	27.7
sea mullet; *n* = 3	*Mugil cephalus*	recreational lake	7 September 2009	Muscle ^D^	3.07 (2.81, 3.83)	6.1	12.7 (11.6, 15.8)
				Liver ^D^	4,740 (4,150, 5,180)	5.4	17,200 (15,000, 18,800)
				stomach contents ^D^	ND		
unidentified mullet; *n* = 2	Mugilidae—undifferentiated	recreational lake	7 September 2009	Muscle ^D^	ND		
				Liver ^D^	963, 1,150	3.9	3,490, 4,170
freshwater shrimp; *n* = 3	unidentified	recreational lake	7 September 2009	whole animal; *n* = 3 specimens combined and extracted as a single sample	ND		
unidentified mullet; *n* = 1	Mugilidae—undifferentiated	reference lake	7 September 2009	muscle	ND		
				liver	ND		
				stomach contents	ND		
sea mullet (*n* = 2)	*Mugil cephalus*	recreational lake	29 September 2009	muscle ^D^	1.94 (*n* = 1; *n* = 1: ND)	12.6	8.02
				liver ^D^	5,230, 7,230	3.7	18,900, 26,200
				stomach contents	4.50, 13.4		
unidentified mullet (*n* = 2)	Mugilidae—undifferentiated	recreational lake	29 September 2009	Muscle ^D^	ND		
				Liver ^D^	1,320, 1,370	4.1	4,780, 4,960
				stomach contents	3.20, 3.49		
sea mullet (*n* = 1)+ goldspot mullet (*n* = 3)+ unidentified mullet (*n* = 1)	*Mugil cephalus* + *Liza argentia* + Mugilidae—undifferentiated	reference lakes	29 September 2009	muscle	ND		
				Liver ^D^	ND		
				stomach contents	ND		

ND: not detected; ^T^: samples analysed in triplicate; ^D^: samples analysed in duplicate.

## 2. Results

### 2.1. Systematic Review of Nodularin in Edible Fish, Shellfish and Crustaceans

Table 1 presents the highest measured concentrations of nodularin in whole organisms and/or selected organs and tissues for each study cited. Error estimates are not considered, as not all reports specified what such estimates represented. Data are presented in increasing order of nodularin concentration. Publications that used ELISA as the sole analytical method generally reported their results in terms such as “total cyanobacterial hepatotoxins” or “microcystin-LR equivalents”, as the ELISA kits used in most studies detect and quantify the ADDA moiety common to microcystins and nodularin. However, the studies in which ELISA alone was used are included in this table because reported exposures were to nodularin-producing cyanobacteria (*Nodularia spumigena*), *i.e.*, exposure to microcystins or microcystin-producing cyanobacteria would appear to be unlikely or minimal.

**Figure 2:**
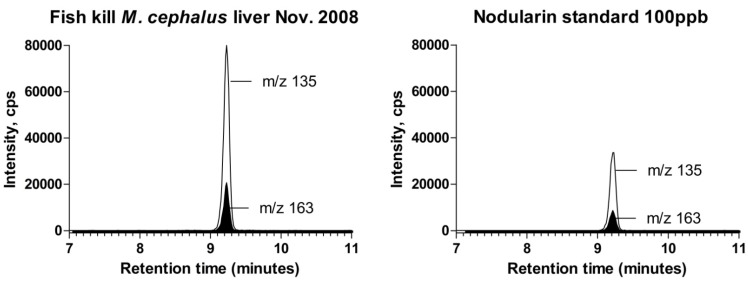
(**a**) Reconstructed partial chromatogram of a diluted methanol extract of a sea mullet liver; (**b**) Reconstructed partial chromatogram of a 100 μg/L nodularin standard solution. Traces shown are for the two transitions monitored, 825.5→135 and 825.5→163. Note the restricted run time (7–11 minutes) plotted on x-axes, employed here to broaden the peaks in order to facilitate visual interpretation.

### 2.2. Fish Species and Measured Nodularin Concentrations in Various Tissues

Nodularin concentrations measured in liver, muscle, peritoneal and stomach contents are presented in Table 2. Common fish names are as defined by the Australian Fish Names Standard [33]. Coefficients of variation for replicate analyses were calculated for nodularin-positive samples. Reported concentrations are not adjusted for recoveries. Microcystin-LR was not detected in any tissues. Figure 2 presents reconstructed partial chromatograms of nodularin product ions.

### 2.3. Spike Recoveries

Table 3 presents recoveries from control fish tissues spiked with nodularin and MC-LR standards. Tissues were spiked in triplicate.

**Table 3:** Spike recoveries.

Matrix	Analyte	Recovery (%) median (range)	C.V. (%)
Liver	Nodularin	83.9 (81.7, 84.0)	1.53
Liver	MC-LR	73.8 (73.1, 75.2)	1.42
Muscle	Nodularin	80.9 (73.7, 84.9)	7.12
Muscle	MC-LR	73.8 (72.4, 76.6)	2.84

**Figure 3:**
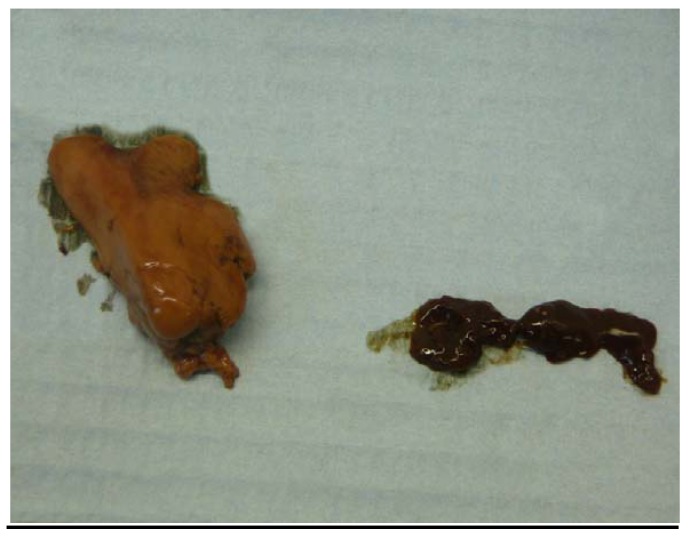
Mullet livers, fish captured 7 September 2009. Left: Partial liver from study lake specimen. Right: Entire liver from control lake specimen. While the liver of the study lake specimen appeared to be grossly enlarged at necropsy, the two livers presented here are from fish of different size classes, so this image should not be used to draw conclusions on relative liver size.

### 2.4. Gross Appearance of Livers; Liver Histology

**Figure 4:**
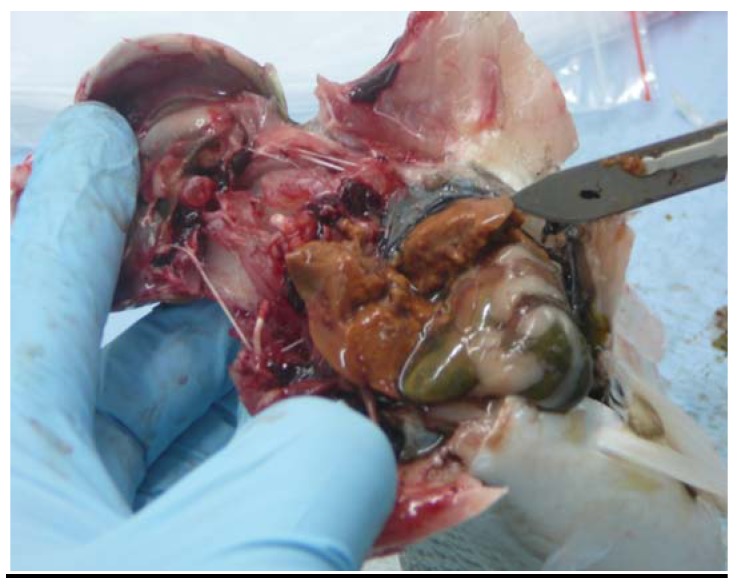
Removal of mullet liver, study lake capture 7 September 2009. These livers were extremely friable; some broke apart when manipulated. Care was needed in order to remove livers *en bloc*.

The livers of all five mullet captured from the study lake on 7 September 2009 were abnormal in appearance: gross hepatomegaly, orange-brown discolouration, and extremely friable. The liver of the single mullet captured at a nearby “control” lake on that day was a dark brown colour, which was obviously different to that of the livers of the study lake fish from which nodularin residue was subsequently detected (see Figure 3). Figure 3 and Figure 4 demonstrate the abnormal colour of mullet liver tissue seen on the 7 September capture. 

Table 4 shows relative liver weights (a.k.a. hepatosomatic index, HSI, *i.e.*, liver weight as a proportion of whole body weight). 

**Table 4:** Relative liver weights of mullet captured on two occasions from the study lake and nearby reference lakes.

Site	Date	*n*	Relative liver weight (%); median (range)	Mann-Whitney U statistic (two-tailed p)
Study lake	7 Sept 09	4	1.66 (1.17, 2.15)	0.0 (0.016)
Control lake	7 Sept 09	1	0.45	
Study lake	29 Sept 09	3	0.93 (0.93, 1.08)	2.0 (0.23)
Control lake	29 Sept 09	4	0.84 (0.67, 0.95)	

### 2.5. Histopathology Examination

Liver sections from study lake mullet caught on 7 September 2009, *i.e.*, with hepatomegaly, orange discolouration and nodularin present (*n* = 3), and 29 September 2009 (no gross abnormality at necropsy but containing nodularin; *n* = 3) and from control lake sites (livers macroscopically normal, nodularin not detected; *n* = 5) were examined, but the results were inconclusive. Findings of the examination are presented in Table 5; because specimens were not fixed under ideal conditions, and other organs—particularly gills, kidney and spleen—were not available for inspection, we have not attempted to interpret these findings in the context of the main focus of this report, that of the chemical analysis of nodularin in mullet tissues. 

**Table 5:** Blinded histopathology examination of livers from the study lake and nearby “control” lakes. Fish captured on two occasions in September 2009.

Slide number	Nodularin (+/-)	Gross appearance of liver	Histopathology	Histopathology Relative Score (HRS)
P19	ND (“control” lake–west pond)	normal	Normal hepatocytes	7–8
P20	ND (“control” lake–model yacht pond)	normal	Melanomacrophages (MMCs) which are darker staining, some pyknotic hepatocytes	5
P21	ND (“control” lake–model yacht pond)	normal	Dark, pyknotic hepatocytes with some enlarged nuclei, increased hepatocellular vacuolation. Liver section appears to dehisce, a possible artefact due to suboptimal fixation but not autolytic.	4–5
P14	ND (“control” lake–west pond)	normal	Mostly normal hepatocytes, with a small number having pyknotic nuclei. MMCs with darkly staining cells. Single granuloma lesion.	7
P16	1.3 mg/kg	normal	Poor fixation resulting in severe autolysis. Precludes useful interpretation.	No score
P17	7.2 mg/kg	normal	Thick fibrous capsule of liver section. Dark staining MMCs. Free macrophages evident. Normal hepatocytes. Eosinophilic granulocytes present. Hyperaemia. The liver appears reactive in the absence of any obvious pathogens.	8.5
P18	1.4 mg/kg	normal	Significant autolysis in areas with dispersal of MMCs, and free macrophages. Precludes useful interpretation.	No score.
P10A	4.7 mg/kg	enlarged, orange/brown colour, friable	Liver section appears to dehisce, a possible artefact due to suboptimal fixation but not autolytic. Pyknotic hepatocytes, with advanced degeneration & necrosis. Absence of MMCs.	3
P10B	5.2 mg/kg	enlarged, orange/brown colour, friable	Pyknotic hepatocytes with occasional normal hepatocytes containing glycogen. Absence of MMCs.	6
P10C	4.1 mg/kg	enlarged, orange/brown colour, friable	Normal hepatocytes generally, although there was some loss of hepatocytes in section. Hepatocytes with glycogen storage and fat vacuolation in some areas. Macrophages present. Absence of MMCs.	6–7
P10D	ND (“control” lake–west pond)	normal	Autolytic changes. MMCs with increased reaction and darker staining cells. Precludes useful interpretation.	No score

HRS criteria: 0–3, Very severe liver changes; >3–5, Severe liver changes; >5–7, Moderate liver changes; >7–10, Mild liver changes to normal; Average HRS for control fish and nodularin exposed is the same at 6.0.

In brief, the gross appearance of livers and histopathology examination suggests that the sampled fish had a variety of liver changes. Whether this is due to nodularin exposure and bioaccumulation cannot be ascertained from this study. A program of laboratory-based studies is indicated in order to investigate dose-response and time-series effects of exposure to nodularin, where environmental variables and age or class size can be controlled.

### 2.6. Stomach Contents

**Figure 5:**
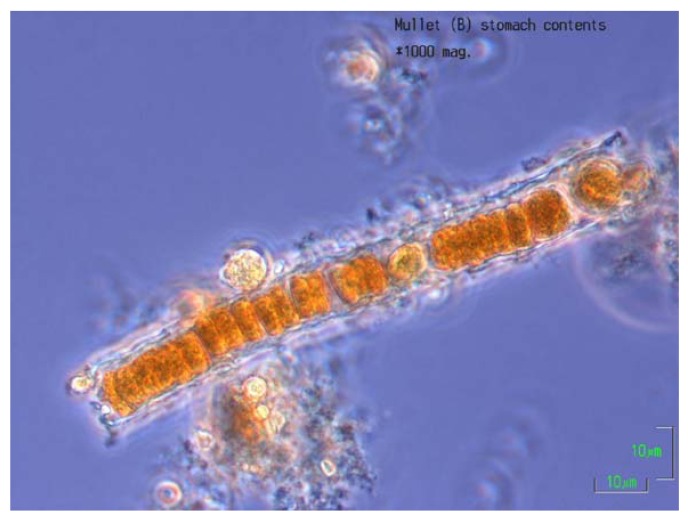
*Nodularia* trichome fragment from the stomach contents of a sea mullet (*Mugil cephalus*), study lake fish kill, November 2008.

Figure 5 is a photomicrograph taken during examination of the stomach contents of a mullet recovered after the initial fish kill at the study lake. Remnants of *Nodularia* trichomes could not be identified microscopically in stomach contents of fish captured in September 2009, however nodularin was found in the stomach contents of mullet caught at the study lake on the second fish capture excursion, on 29 September 2009 (see Table 2).

### 2.7. Comparison of Wet Weight *vs.* Dry Weight Yields for Nodularin

Tissue dry weight equivalents presented in Table 2 were calculated from average moisture content of lyophilized tissues. Mullet liver contained 72.4% water (C.V. of duplicate measurements = 1.06%); muscle tissues of mullet recovered after the fish kill contained 69.1% water (C.V. 0.44%) and muscle tissue of captured mullet contained 75.8% water (C.V. 0.69%). 

## 3. Discussion

This investigation has demonstrated that mullet are capable of accumulating high concentrations of nodularin in their livers. Mullet delivered to our laboratory following a fish kill in November 2008 had the highest liver nodularin concentrations detected in this investigation, with a median concentration of 12 mg/kg wet weight, equivalent to 43.5 mg/kg dry weight (Table 2). Indeed, these levels appear to be higher than concentrations found in edible aquatic and marine animals by natural exposures reported elsewhere in the world. Table 1 shows that higher concentrations of nodularin were seen only in bivalve molluscs exposed to nodularin under experimental conditions in the laboratory. Those concentrations—between 14 mg/kg and 80 mg/kg in whole soft tissues [29,30,31,32], and 245 mg/kg in mussel digestive gland [31,32]—presumably included significant proportions of nodularin in undigested form present in alimentary tracts. The highest nodularin concentration in fish liver reported elsewhere was 2.2 mg/kg, from Baltic Sea flounder [28]. Nodularin concentrations in fish muscle measured in this study were lower than levels reported elsewhere: we found a maximum concentration of 57 μg/kg in mullet, compared to maximum reported levels of 200 μg/kg in roach muscle [16]. 

An intriguing finding arising from this field investigation is the difference in nodularin concentrations across the different fish species recovered from the study lake after the initial fish kill. We found only low levels of nodularin in greasy rockcod and longfin eel livers (91 μg/kg and 212 μg/kg respectively), and we did not detect the toxin in muscle extracts from these fish. We could not recover sufficient liver tissue for analysis from two specimens of bream; nodularin was not found in the flesh of these fish. We suspect that the remarkable difference in nodularin concentrations across these species can be explained both by the chemistry of the toxin and the feeding ecology of these fish. Nodularin is highly water soluble; it can move up different trophic levels, but does not appreciably biomagnify. Mullet feed by dredging up sedimentary mud, from which they extract nutrients. These fish are able to digest microalgae, cyanobacteria, terrestrial plant debris, and small gastropods and copepods through the grinding action of sand particles in the highly muscular stomach [34]. This feeding strategy would thus appear to place mugilids at high risk of exposure to and uptake of cyanobacterial toxins. 

The study lake experienced a dense nodularin-producing bloom of *Nodularia spumigena* in September 2008. Phycological investigations and interventions addressing the direct public health risks to recreational users are detailed in the accompanying paper by McGregor *et al*. [35]. Investigations into the bioaccumulation of nodularin in resident fish were initiated after a fish kill that followed treatment of the lake by Phoslock^®^, a patented lanthanum modified clay product designed to sequester phosphate from the water column and sediment pore water and thus make phosphorus unavailable to cyanobacteria blooms [36,37]. While we have found very high levels of nodularin in mullet livers, particularly after the fish kill in November 2008, we do not suggest that acute nodularin intoxication was responsible for the fish mortalities. Given the temporal sequence of events related to Phoslock^®^ treatment, *i.e.*, *in situ* application followed by bloom collapse after some two weeks, followed by the fish kill, we suspect that increased heterotrophic bacterial activity from decomposition of cyanobacterial biomass resulted in deoxygenation of the water column. So the engineered rapid bloom senescence is a more likely explanation for this mass mortality than the high concentrations of nodularin found in mullet livers. Supporting this supposition is the observation that other species were involved in the fish kill, but relatively low concentrations of nodularin were detected in their livers; only mullet had high hepatic nodularin loads. Mullet retrieved from the study lake on all three occasions (the fish kill and two capture excursions) had been feeding, as sediment was found in their stomachs, which further supports the supposition that bioaccumulated nodularin was not responsible for the mass mortalities. *Nodularia* can produce other toxic compounds, usually described as minor variants of nodularin [38], as well as allelopathic properties unrelated to nodularin [39]. 

Mullet captured from the study lake in September 2009 were found to contain nodularin in muscle and liver tissues; while nodularin concentrations in liver were lower than seen in earlier fish kill specimens (see Table 2), they were high in comparison to levels reported in fish livers elsewhere in the world (Table 1). Mullet caught by hand seine from the study lake appeared to behave normally, *i.e.*, they would vigorously leap over the net to escape when the seine was gathered. We assume that the presence of nodularin in these fish some 10 months after the initial bloom in late 2008 is the result of this recreational lake having experienced sporadic, ephemeral blooms of toxic *N. spumigena* in the interim, although the possibility of longer-term exposure to *Nodularia* and/or nodularin immobilized in lake sediments since the initial bloom must be considered. Water samples collected at the time of the fish capture excursions in September 2009 contained only low levels of *Nodularia*. 

### 3.1. Evidence for Different Mullet Species in the Study Lake

Of the five mullet caught in the study lake on 7 September 2009, three specimens were identified as *Mugil cephalus*, whereas two were mullet species that could not be matched to the BOLD database. These two unidentified mullet belonged to the same species, but we were unable to determine exactly which species it was. Interestingly, the nodularin profile varied across these different mullet species. While the sample numbers here are small (*n* = 3 and *n* = 2), thus demanding caution in the interpretation of these findings, the three *M. cephalus* specimens had essentially identical nodularin concentrations in their livers (median 4.7 mg/kg wet weight) and nodularin was detected in the muscle of these fish (median 3.1 μg/kg). By contrast, the hepatic nodularin concentrations in the two unidentified mullet were substantially lower (0.96 mg/kg and 1.2 mg/kg) than those found in the sea mullet; nodularin was not detected in the muscle. This pattern was repeated on the second mullet capture excursion on 29 September. Of the four mullet caught that day from the study lake, DNA sequence analysis showed that two specimens were *M. cephalus*, and the other two were an unidentified mullet of the same species. Further examination of nucleotide sequences from the unidentified mullet caught from the study recreational lake on both days in September 2009 has shown that these four specimens belong to the same species. Thus we have evidence for two mullet species inhabiting this lake: *M. cephalus* and an unknown mugilid. Of these two species, *M. cephalus* consistently had higher hepatic nodularin levels than the unidentified mullet. Differences in wet weight liver nodularin concentration between both species were statistically significant with a low-power non-parametric test (Mann-Whitney U statistic: 0.0, two-tailed *p* = 0.016). So we appear to have found variation in tissue nodularin concentrations between different mullet species. While variable nodularin uptake and/or elimination kinetics across individual Mugilidae species may explain these results, we suggest that different feeding strategies and resource partitioning should be priority avenues for inquiry in this regard. The evolutionary diversity of the Mugilidae is centred on the mouth parts; different mullet species display preferences for specific inorganic particle size in the substrate [34,40]. Mullet species are reported to select specific particle sizes with a pharyngeal filter apparatus, though choice of substrate may also contribute to observed species-dependent variability in particle size in the stomach. Thus individual mullet species will partition the food resource on the basis of inorganic particle size preference [34,41]. A mosaic distribution of *Nodularia* across surface sediments of the study lake, and species-specific substrate preferences may be factors worth investigating in order to explain these provisional findings of inter-species variability in tissue nodularin levels within the Mugilidae. 

### 3.2. Mullet Livers: Macroscopic and Microscopic Findings

Livers from mullet captured at the study lake on 7 September 2009 (*n* = 5) were an orange/tan colour, and appeared to be enlarged at necropsy. The relative liver weights presented in Table 4 support the visual assessment of hepatomegaly in study lake fish from 7 September 2009 when compared to relative weights of control lake mullet livers (*p* = 0.016). However, that observation did not hold for mullet caught at the study lake some three weeks later. Livers at that time were a more normal-looking brown colour, and did not appear enlarged. Again, relative liver weights of 29 September 2009 fish were not significantly larger as a group compared to those of fish from the nearby reference lakes (*p* = 0.23). Yet we found no obvious correlation between liver nodularin concentration and gross appearance of the livers; while there appeared to be a species relationship to liver nodularin concentration, as discussed above, nodularin concentrations measured in both species were higher on the second capture day (29 September 2009) when livers were macroscopically normal. Livers from the control lake fish did not contain detectable nodularin but controlled studies are required to ascertain whether liver discolouration in mullet is a reliable indicator of exposure to nodularin, and this will require a better understanding of the normal range of liver colouration in mugilids. We considered the possibility that our findings of hepatomegaly and orange/tan discolouration of the liver were related to exposure to *Nodularia* and/or nodularin, but we were unable to find reports that supported this hypothesis, as macroscopic changes in fish livers are not described in the literature on nodularin exposure in fish (Table 1). Kankaanpää *et al*. [21], in their oral dosing study of nodularin in trout, reported no gross pathological changes apart from inflammation of the intestine. From the larger body of literature on the effects of microcystins on fish, reviewed by Malbrouck and Kestemont [42], varying descriptions of gross pathological presentation of the liver are found. Kotak *et al*. [43] describe livers that were “wet, soft and pale tan or creamy white, with multiple, small ecchymoses visible beneath the capsule” in rainbow trout (*Oncorhynchus mykiss*) dosed with MC-LR at 1 ppm, and “red/tan mottled livers” seen at lower exposures. Best *et al*. [44], also working with *O. mykiss*, describe swollen, pale grey-coloured livers in fish exposed to MC-LR. Hepatomegaly, as reported by the hepatosomatic index (HSI), is also reported by these same authors. Kotak *et al*. [43] noted dose-related increases of 39% and 45% in HSI of MC-LR-dosed trout compared to controls; Best *et al*. [44] reported increased HSI of approximately 50%. In this study, the median HSI in mullet caught on 7 September 2009 was 1.66, a 52% increase over the median “control” group HSI of 0.8 (*p* = 0.016). A histological finding (Table 5) that correlated with the macroscopic examination of livers was that the three fish with gross hepatomegaly and orange discolouration seen at necropsy had a markedly reduced number of melanomacrophage centres (MMCs) in liver tissues; all other specimens, *i.e.*, fish from control lakes and fish from the study lake caught on 29 September 2009—the latter with nodularin present but macroscopically normal livers—had clearly identifiable MMCs in liver tissue. Fish MMCs are macrophage aggregates, found in splenic and renal tissues and in the livers of some groups [45]. 

Because fish liver specimens were not prepared under ideal conditions, *i.e.*, they were not removed and formalin fixed in the field immediately after capture, some autolysis was evident in three of 11 slides examined. However, fish were placed on ice at the field site, which presumably slowed down autolytic changes sufficiently for some histological assessment to be made. Histological examination of liver tissues not affected by autolysis could not differentiate specimens that were exposed to nodularin from those captured from the reference lakes with no nodularin liver residues (both fish groups were independently allocated a HRS of 6.0). 

In future exposure studies, organs in addition to the liver such as gills, kidney, spleen, central nervous system (brain, spine), gastrointestinal tract, gonads and muscle should be collected for histopathology and nodularin residues. Collectively, examination of these tissues may provide important information on the toxicokinetics of nodularin in the fish and will allow us to determine whether nodularin produces recognisably consistent pathological changes. 

Future use of organ histopathology in the study of the pathogenic toxicology of nodularin in mullet would need to consider the exclusion of water quality factors not related to nodularin, e.g., other xenobiotic pollutants. The reason being that liver and organ responses to toxicants are generally non-specific, such that concurrent exposure to toxicants or poor water quality may obscure changes due to nodularin exposure alone. For this investigation, we measured only *Nodularia* and nodularin in water from the study lake and reference lakes, and in tissues of fish captured from these sites. Field studies in closed water bodies where a broader range of water quality parameters are measured for both reference and affected sites may aid in the interpretation of liver and organ changes where the risk of interest is nodularin or other cyanotoxins.

Our findings in this regard bear similarities to those reported by Kankaanpää *et al*. In that study, nodularin was measured in livers of flounders captured from the Gulf of Finland; histological examination of livers was also conducted. A range of pathological changes were seen that could not be correlated with nodularin concentration. Lesions seen in their wild-caught fish were attributed to a variety of probable causes such as parasite infestation and exposure to anthropogenic toxicants [28]. 

### 3.3. No Evidence for Post-Mortem Redistribution of Nodularin

We considered the possibility of post-mortem redistribution of nodularin, given that fish sent to us after the fish kill in November 2008 were in poor condition; some specimens presented with obvious early decomposition of internal organs. The likely sequence of events following (presumed) death due to deoxygenation resulting from increased heterotrophic bacterial activity associated with the senescent *Nodularia* bloom is that dead fish would have sunk to the lake bottom, where they may have remained for a day or more before buoyancy due to gases produced during decomposition made the fish kill apparent. Post-mortem redistribution of pharmaceuticals is a concern for forensic toxicologists; lipophilic compounds are more prone to redistribution, which follows a concentration gradient as tissue breakdown occurs [46,47]. While nodularin is a water soluble toxin, we were nonetheless *concerned* about the possibility that undigested toxin present in the alimentary tract of the fish-kill mullet may have contributed to the high concentrations of nodularin we detected in hepatic tissues of these fish. We extracted peritoneal contents of these three specimens, but peritoneal nodularin concentrations were well below those found in liver tissues (median 0.2 mg/kg *vs.* 12 mg/kg wet weight; see Table 2). We conclude, therefore, that the toxin we detected in the livers of these mullet was concentrated in those tissues through dietary exposure and active uptake, as the toxicokinetics and organ-specific affinities are understood for the ADDA-containing cyanotoxins [48,49]. 

### 3.4. No Evidence for Biomagnification of Nodularin

Nodularin concentrations in carnivorous fish were several orders of magnitude lower than those found in the two species of Mugilidae. This supports the general premise outlined by Ibelings and Havens [50] that for water-soluble cyanotoxins such as nodularin, biodilution rather than biomagnification can be expected at higher trophic levels. Our work also supports the findings and conclusions of Berry *et al*. [51] regarding bioaccumulation in fish of the microcystin (MC) group of cyanotoxins. Berry *et al*. found higher concentration of MCs in phytoplanktivorous fish compared to omnivores and zooplanktivores, with the implication that bioaccumulation of these water-soluble toxins is more a function of direct dietary exposure to cyanobacterial cells, not a biomagnification effect. The discovery that mullet are capable of concentrating extraordinarily high concentrations of nodularin in hepatic tissues may be explained both by a presumed congruity in uptake kinetics with the cyclic peptide microcystins, these utilising a process of active transport into the liver [52], and a high degree of exposure to *Nodularia* and hence nodularin through their feeding strategies.

### 3.5. Sources of Nodularin Found in Mullet Tissues

Public health authorities and researchers first became aware of a dense *Nodularia* bloom at the study lake in September 2008. As discussed above, the fish kill some two months later followed treatment of the lake with Phoslock^®^. We thought that a deoxygenation event triggered by collapse of the bloom was a more likely explanation for the fish kill than a direct toxicity effect due to nodularin, in part because fish other than mullet involved in the kill had much lower concentrations of nodularin in their livers at the time. On two occasions three weeks apart in September 2009, we again found high concentrations of nodularin in the livers of mullet captured from the study lake. Lake water at the time of the two fish capture surveys in September 2009 was free of detectable nodularin, and *Nodularia* populations were low. We did, however, receive anecdotal reports of a green discolouration in the lake some two weeks before our first capture survey on 7 September 2009. We assumed that the findings of high nodularin levels, enlarged and discoloured livers in early September, followed by high nodularin levels and normal-looking livers three weeks later could be explained by re-exposure to and partial recovery from nodularin produced by a transient *Nodularia* bloom in late August 2009. We suspect that this lake is intermittently affected by phytoplankton blooms; a review of satellite images of the site since 2003 using the historical imagery function in Google Earth suggests a dynamic process in this regard. However, we cannot rule out the possibility that nodularin may have remained intact in the lake sediment since the 2008 bloom, and may have been available to mullet—again, through their unusual feeding strategy involving uptake of sediment material—for subsequent exposure through the following year. Some organic compounds produced by cyanobacteria and other phytoplankton are extremely stable in sediments. Indeed, chlorophyll, carotenoids and other compounds can be reliably detected in prehistoric sediments and are used as the basis for determining phytoplankton assemblages in ancient lakes [53,54]. Nodularin is reportedly capable of persisting for “several months” in sediments [12], and the related cyclic peptide toxins, the microcystins, have been recovered from lake sediments at a depth of 30cm, which implies a capacity for longevity in some circumstances [55]. Experimental work has shown that nodularin can adsorb to sediment particles [56].

### 3.6. Public Health Implications

The discovery of nodularin in liver and muscle tissues of mullet has implications for public health, with the potential for dietary transfer of the toxin should these fish be caught and eaten. Nodularin in marine food products clearly concerns researchers in Baltic Sea states, as seen in the literature cited in Table 1. Other cyanotoxins are also coming under scrutiny from a food safety perspective, with a growing body of literature reporting the presence of cyanobacterial toxins in foods of marine and freshwater origin. The majority of reports pertain to microcystins; for recent examples see [57,58,59,60]. Microcystins are probably the most frequently encountered and widely distributed group of freshwater cyanobacterial toxins. Saxitoxins (of cyanobacterial origin) and cylindrospermopsin have been found in edible marine and aquatic animals after both natural and experimental exposures [61,62]. 

The emerging public health issue of cyanobacterial toxins in fish and shellfish was discussed by Ibelings and Chorus [63], who present a three-tier guideline value proposal for microcystin-LR in aquatic foods. These authors have calculated tolerable exposure levels for both children and adults, through acute, sub-acute and chronic exposures. The Australian state of Victoria has produced guideline concentrations for a range of cyanotoxins in seafood and shellfish harvested from the Gippsland Lakes [64]. This estuarine lake system has long been adversely affected by toxigenic cyanobacterial blooms, principally *Nodularia*. To our knowledge, Victoria is the first government agency anywhere in the world to move beyond *ad hoc* decision-making to direct fisheries closures for protection of public health from toxic cyanobacteria by adopting and publishing guideline values developed from a human health risk assessment approach. However, governments across the world are recognising the risks these potent toxins present, and have sanctioned guidelines for exposure from drinking water and recreational waters [65]. Many national and trans-national agencies (the European Union, for example) monitor and regulate concentrations of eukaryotic marine algal toxins such as saxitoxins, okadaic acid and domoic acid in seafood and shellfish [66]. Cyanobacterial toxins are considered along with eukaryotic HAB toxins in Denmark, where commercial harvesting of mussels is subject to closure by regulatory authorities in the event that toxins are detected through their monitoring program [65]. Victoria has assumed a food safety leadership role with regard to monitoring and regulation of cyanotoxins in foods harvested from a lake system frequently subject to toxic cyanobacteria blooms. Their guidelines now form the basis for specific public health advice by the Victorian Department of Health regarding consumption of seafood and shellfish from the Gippsland Lakes; results of their monitoring program are posted on a publicly accessible website [64,67]. 

A direct application of the Victorian Government guidelines to the data generated for this report should be interpreted cautiously, as the guidelines ascribe recommended safety values to cyanotoxin concentrations in whole fish [64], which we did not measure (we analysed muscle and liver tissues separately). Nodularin concentrations in mullet muscle were all below the guideline values of 39 ppb for adults and 24 ppb for children. But concentrations in liver were noteworthy: the highest wet weight liver concentration we found in fresh mullet, 7.2 ppm, represents a 185-fold exceedance for adults and a 300-fold compliance failure for a child. 

In Australia, toxic *Nodularia* blooms have affected large estuarine systems in the southern and south-west regions of the continent. The Murray River’s estuarine lakes in South Australia, Western Australia’s Peel-Harvey and Vasse-Wonnerup estuaries and Victoria’s Gippsland Lakes all suffer massive intermittent *Nodularia* blooms; shellfish and prawn fisheries in the Gippsland Lakes have been subject to temporary closure [13,68,69,70,71,72]. *Nodularia* blooms have declined in the Harvey estuary since the construction in 1994 of an artificial channel to flush the system with seawater, though blooms still occur in tidal reaches of inflowing rivers. *Nodularia* blooms have also been reported from a coastal lake system in Tasmania, Australia’s most southerly State [73]. The Eastern seaboard (and lower latitude) state of New South Wales has not, to our knowledge, experienced any significant *Nodularia* blooms in its estuaries, and in Queensland this species has only been found in the recreational lake that is the focus of this report and its companion paper [35]. 

Various mullet species, principally *M. cephalus*, are harvested commercially and recreationally from estuaries in all Australian mainland states; the *Nodularia*-prone Peel-Harvey, Vasse-Wonnerup, Gippsland Lakes and Murray Lakes systems support valuable commercial mullet fisheries [72,74,75,76]. Australia’s wild-caught mullet fisheries are valued at more than A$14 million annually, with production of 5,500 tonnes in 2009–2010 [77]. Globally, various mullet species support commercial, recreational and subsistence fisheries. Mullet aquaculture and larviculture are very substantial industries worldwide [78,79]. Egypt produced over 150,000 tonnes in 2005, which then represented 29 per cent of that nation’s aquaculture production [80]. *M. cephalus* are being investigated for their potential as a mariculture bioremediator; benthic enclosures anchored beneath finfish net cages are stocked with mullet, which feed on the sediments with accumulated organic waste that falls out of the main enclosures [81,82]. 

We suggest that Mugilidae raised in polyculture systems or as bioremediators in nutrient-enriched aquaculture or mariculture processes that may facilitate proliferation of cyanobacterial blooms, should such stock enter the human food supply, should be subject to research scrutiny for their ability to accumulate cyanotoxins such as nodularin, microcystins and cylindrospermopsin. This study presents preliminary evidence that mullet have an unusual capacity to concentrate such toxins in their livers, and that the toxins may also be present in muscle tissues. Concentrations of cyanotoxins in mullet tissues that may pose a food safety risk may not manifest in obvious behavioural changes in the fish. 

Viewing the high concentration of nodularin in mullet livers from a food safety perspective, the risk of acute intoxication arising from this particular event would appear to be somewhat remote, given that fish liver is generally not eaten by the dominant Caucasian population in Australia, and to capture mullet from this small recreational lake would have required a fairly concerted effort to trawl with nets during the night, as the proprietor of the cable ski business does not allow fishing activities when the park is operating in daytime hours. However, the potential for acute poisoning due to cyanotoxins in mullet liver should be considered; dietary practices within some communities, particularly those of island cultures for which seafood is of paramount significance, suggest fish liver and other viscera are consumed. Fish liver is a delicacy in Micronesia; indeed, nutritional research in these island states seeks to encourage consumption of fish liver as an excellent source of vitamin A [83]. As for mullet, we have anecdotal reports that sea mullet liver is a constituent of certain dishes prepared by particular ethnic communities in Australia. A paper from a Spanish group notes that the mugilid *Liza ramada* “…may be consumed with or without the liver, depending on eating habits” [84]. The internet is generously supplied with recipes for mullet liver, but these appear to refer to “red mullet”, a common name that encompasses several Mediterranean species not belonging to the Mugilidae. Liver of European flounder is regarded as a delicacy by certain cultural groups; bioaccumulation of nodularin by flounder is the focus of attention by researchers in Baltic Sea states, and recommendations have been made in this regard to avoid consumption of flounder liver [28].

Mullet roe is a profitable export commodity [85], including from Australia where roe is the most valuable component of the fish [86,87]. Future toxicokinetics studies of nodularin and microcystins in mullet will need to assess the capacity of these toxins to bioaccumulate in the roe.

## 4. Methods

### 4.1. Systematic Review of the Literature: Nodularin in Seafood and Shellfish

A systematic review of the literature was conducted to identify observational and experimental measurements of nodularin in aquatic and marine animals that are food items for human consumption. The search string “nodularin AND (fish OR seafood OR shellfish)” was used in both PubMed and Web of Science bibliographic databases. Manuscript titles and abstracts were perused to identify those reports discussing measurement of nodularin in tissues of seafood products. The bibliographies of papers thus identified were reviewed in order to identify other published reports that appeared to meet inclusion criteria for Table 1. Only English-language papers were consulted; the grey literature was not searched. Several papers describing nodularin concentrations in the three-spined stickleback (*Gasterosteus aculeatus*), a species often used for laboratory-based experiments, were not included as this fish is not a common food item, though it has been commercially harvested in previous decades and processed into fishmeal and animal fodder [88].

### 4.2. Fish Specimen Collection

Liver and muscle tissues were removed from seven specimens collected after a fish kill at the lake under investigation in November 2008. Tissues from three sea mullet (*Mugil cephalus*), two yellowfin bream (*Acanthopagrus australis*), one longfin eel (*Anguilla reinhardtii*) and a greasy rockcod (*Epinephelus tauvina*) were examined. The fish kill occurred 12 days after commencement of a treatment regime comprising Phoslock^®^ (a phosphorus sequestrant) and polyaluminium chloride (a flocculant) adopted in an attempt to mitigate a toxic bloom of *Nodularia* that occurred initially in September of 2008. Another eel was captured in April 2009, *i.e.*, several months after the bloom had dissipated. Tissues for control purposes were provided by two seafood processors: sea mullet livers and fillets from A.J. Raptis & Sons, Colmslie, Queensland (sourced from a fishery offshore from Ballina, New South Wales) and a whole longfin eel was purchased from Manchester Eels, Coopers Plains, Queensland.

Specimens retrieved from the study lake in November 2008 after the fish kill were undergoing early decomposition, so macroscopic and microscopic assessment of the condition of internal organs was not possible. Liver tissue was identifiable and able to be removed in sufficient amounts for extraction and quantification of nodularin in all specimens except for the two bream; muscle tissue only was examined in those fish.

Subsequent field investigations focused on mullet, as analysis of tissues from the fish kill showed that these species accumulated much higher concentrations of nodularin compared to other fish collected at the time. Field excursions to capture mullet from the cable ski park and nearby lakes were undertaken on two days in September 2009. On 7 September, a combined approach using shore seine netting and vertical gill netting was used. A subsequent excursion to capture fish from the study lake, and in particular to acquire more specimens from the nearby control lakes, was conducted on 29 September. The seine method proved to be more successful for catching mullet, so was employed exclusively on the follow-up capture excursion on 29 September. Captured mullet were immobilised by placing in ice slurry, after which they were killed by rapidly severing the spinal cord with a sharp knife. Intact carcases were kept on ice at 4 °C until necropsies were conducted the following day. 

The recreational lake that is the focus of this investigation—a cable ski park in Logan Shire, southeast Queensland—is referred to as the “study lake,” or “recreational lake” throughout this report. Properties located approximately 1.5 km NW of the cable ski park, occupied by a golf course and a sand processing facility, contain a series of artificial freshwater and brackish water lakes. Two of these lakes—“west pond” and “model yacht pond”—were fished for mullet to serve as reference specimens. These sites are referred to collectively as “control lakes” or “reference lakes” in this report. 

### 4.3. Detection and Measurement of Nodularin in Fish Tissues

Of the fish received after the 2008 fish kill, the longfin eel was in the best condition; the liver of this specimen was used to develop the most suitable extraction method. We initially used a methanol extract with hexane wash and solid-phase extraction clean-up, but a simple methanol extraction (in duplicate) followed by HPLC-MS/MS in multiple reaction mode was found to be a more effective and reproducible technique, with minimal matrix effects observed in the electrospray mode. 2.0 mL of 80% v/v methanol in chromatography-grade water was added to 1 g tissue (liver and muscle tissues, analysed separately), homogenised with an Ultra Turrax T25 macerator, centrifuged at 3,500 rpm for 20 minutes and the supernatant was decanted. A second extraction was conducted, supernatants were combined and syringe filtered (0.45 μm). Samples were initially analysed in triplicate when sufficient tissue was available; after satisfying ourselves that our method was reproducible, subsequent analyses were conducted in duplicate. 

Nodularin was determined by HPLC-MS/MS using an AB/Sciex API4000Q mass spectrometer (AB/Sciex, Concord, ON, Canada) equipped with an electrospray (TurboV) interface coupled to a Shimadzu Prominence HPLC system (Shimadzu Corp., Kyoto, Japan). Separation was achieved using a 5 micron 150 × 4.6 mm Alltima C_18_ column (Alltech®, Deerfield, IL, USA) run at 40 °C, and a flow rate of 0.9 mL/min with a linear gradient starting at 100% A, ramped to 90% B in 12 minutes then to 100% B in 1 minute and held for 3 minutes followed by equilibration at 100% A for 5 minutes (A = 1% methanol in HPLC grade water, B = 95% methanol in HPLC grade water, both containing 0.1% formic acid). The mass spectrometer was operated in the positive ion, multiple reaction-monitoring mode using nitrogen as the collision gas. Transitions monitored for nodularin were 825.5 (M + H)^+^ to 135 and 163 (relative intensity 28%) using a declustering potential of 125 and collision energies of 75 and 65 electron volts. The transitions monitored for microcystin-LR were 995.6 (M + H)^+^ to 135 and 213.2 (relative intensity 52%) using a declustering potential of 130 and collision energies of 110 and 85 electron volts. The retention times for nodularin and MC-LR were 9.2 and 9.6 minutes respectively.

Positive samples were confirmed by retention time and by comparing transition intensity ratios between the sample and an appropriate concentration standard from the same run. Samples were reported as positive if the two transitions were present, retention time was within 0.15 minutes of the standard and the relative intensity of the confirmation transition was within 20% of the expected value. The value reported was that for the quantitation transition. Using a 10 μL injection volume the limit of detection for this method was typically less than 1 μg/L, with a reporting limit of 2 μg/L in the final aqueous methanol extract. Response was linear to at least 500 μg/L.

### 4.4. Spiking Experiments

Control sea mullet liver and muscle samples were spiked in triplicate with 750 pg nodularin standard and separately with 145 pg microcystin-LR. Standards were dissolved in methanol. Extracts were prepared as described above, and spiked with toxin prior to maceration. Nodularin and MC-LR standards were purchased from Abraxis LLC (Warminster, PA, USA). Nodularin in-house standard was prepared from *N. spumigena* bloom material. Lyophilised, sonicated cells were extracted with aqueous methanol, further concentrated by C_18_ solid-phase extraction, and fractionated by reverse-phase HPLC. The nodularin-containing fraction was quantified by reference to the purchased standard and by UV spectroscopy; this in-house standard was used for spiking and routine analytical quantification. 

### 4.5. Identification of Fish Species

Fish specimens captured from the study and control lakes were identified by DNA barcoding and comparison with the BOLD database, as described in [89]. Specimens that were unable to be reliably identified to species level by this procedure are reported at the Family taxon.

### 4.6. Liver Histology

Livers were removed in the laboratory the day after specimens were captured. Livers that were able to be removed intact from mullet caught on the two fish netting excursions in September 2009 (*n* = 12) were weighed. Liver sub-samples were fixed in 10% buffered formalin, sectioned, stained with haematoxylin & eosin and mounted on glass microscope slides. Slides were labelled with an alpha-numeric code in order to blind pathologists (authors RC and AAS) to the identity of control and study lake fish.

### 4.7. Comparison of Wet Weight *vs.* Dry Weight Yields for Nodularin

Muscle tissues of four mullet specimens from the study lake were weighed in duplicate (9–14 g), frozen and lyophilised (Martin Christ Alpha 2–4 LD, Osterode, Germany). Dry tissues were then reweighed for estimation of dry weight yields. Liver tissues from study fish were not available for lyophilisation; sea mullet livers were donated by A.J. Raptis & Sons, Brisbane, Australia. As for muscle, livers were weighed and lyophilised in duplicate.

### 4.8. Nodularia and Nodularin in Stomach Contents

The stomach of one of the mullet retrieved from the study lake after the initial fish kill in November 2008 was intact and able to be opened. There was insufficient material inside the stomach to perform a solvent extraction for analysis of nodularin, but microscopic examination of the stomach contents revealed numerous fragments of *Nodularia* (see Figure 5). Stomach contents of mullet captured from study and control lakes in September 2009 were examined microscopically, and solvent extraction and HPLC-MS/MS for nodularin detection and quantification was conducted. 

### 4.9. Statistical Analysis

Distribution-free Mann-Whitney tests were used to compare:

1. Relative liver weights of fish caught in the study lake with those of fish captured from nearby control lakes. These are comparisons between study lake HSI on each capture day, *i.e.*, *n* = 4 on 7 September 2009 and *n* = 3 on 29 September 2009, and pooled control lake fish HSI caught on both days (*n* = 5).
2. Wet weight liver nodularin concentrations in *M. cephalus* and the unknown mugilid species captured from the study lake in September 2009.

A non-parametric test was adopted because small sample sizes did not permit normal distribution assessments. Analyses were conducted with GraphPad Prism v5.03; a significance level of 5% (*p* < 0.05) was adopted.

## 5. Conclusions

This observational study of fish inhabiting a recreational lake periodically affected by toxic *Nodularia* blooms has demonstrated that bioaccumulation of nodularin occurred within this isolated, enclosed food web. Detection and quantification of nodularin appears to be reliable and reproducible with negligible matrix interference using a simple methanol extraction without sample cleanup and multiple reaction monitoring by HPLC-MS/MS. Coefficients of variation for replicate analyses were mostly below 10%. Recoveries of nodularin from spiked tissue samples were above 80%. Microcystin-LR was used as a surrogate standard and was recovered at a slightly lower proportion, at 74%. 

We found mullet with high concentrations of nodularin in their liver that did not behave abnormally at capture, where the livers were not visibly diseased or discoloured (gross liver morphology may or may not reflect exposure to nodularin in mullet), and there were no indications of a concurrent cyanobacteria bloom at the time of capture. Therefore, in the absence of any immediate indication of actual or potential ill-health in fish that may be netted under similar conditions, it is entirely feasible that human exposure to nodularin might occur through consumption of mullet. For this reason, we recommend surveillance sampling of food fishes like mullet which coexist with nodularin-producing cyanobacteria blooms. This will be necessary in order to estimate human health risks and develop food safety regimes to protect consumers of fish products that may contain nodularin. 

This work also invites further research enquiry to investigate the toxicokinetics of cyanobacterial hepatotoxins in the Mugilidae. Baseline studies on the dose-related gross pathology and histological changes attributable to nodularin intoxication should complement such enquiries. Laboratory-based studies are indicated in order to control environmental variables. 
